# Supplementary material for: Mental illness and COVID-19 vaccination: a multinational investigation of observational & register-based data
Source: Nat Commun. 2024 Sep 26;15:8124. doi: 10.1038/s41467-024-52342-1 (PMC11427681; doi:10.1038/s41467-024-52342-1)
Supplement: Supplementary file 1 — Supplementary Information [file 41467_2024_52342_MOESM1_ESM.pdf]

Supplementary Material

Mental illness and COVID-19 vaccination: a multinational investigation of observational & register-based data

Mary M Barker[1]*, Kadri Kõiv[2]*, Ingibjörg Magnúsdóttir[3]*, Hannah Milbourn[4]*, Bin Wang[5,6]*, Xinkai Du[7,8]*, Gillian Murphy[1], Eva Herweijer[1], Elísabet U Gísladóttir[1], Huiqi Li[9], Anikó Lovik[1,10], Anna K. Kähler[11,1], Archie Campbell[4], Maria Feychting[1], Arna Hauksdóttir[3], Emily E Joyce[1], Edda Bjork Thordardottir[3], Emma M. Frans[11], Asle Hoffart[7,8], Reedik Mägi[2], Gunnar Tómasson[3,12], Kristjana Ásbjörnsdóttir[3], Jóhanna Jakobsdóttir[3], Ole A. Andreassen[13,14], Patrick F. Sullivan[11,15], Sverre Urnes Johnson[7,8], Thor Aspelund[3], Ragnhild Eek Brandlistuen[6,16], Helga Ask[5,17], Daniel L McCartney[4], Omid V Ebrahimi[18,17], Kelli Lehto[2], Unnur A Valdimarsdóttir[1,3,19], Fredrik Nyberg[9], Fang Fang[1]

*Equal contribution

Table of Contents

Supplementary Table 1: Ethical approvals obtained for each included COVIDMENT cohort study. 3

Supplementary Table 2: Key COVID-19 vaccination dates for countries included in the COVIDMENT study population. 3

Supplementary Table 3: ICD-10 codes used to define mental illness (in the EstBB-C19 and EstBB-EHR cohorts) and physical comorbidity status (in the EstBB-EHR cohort). 4

Supplementary Table 4: Timing of variable definitions in the participating COVIDMENT cohorts. 5

Supplementary Table 5: National average Containment and Health Index from the Oxford COVID-19 Government Response Tracker (OxCGRT) between January 2020-September 2021, and resulting sub-group analysis category 8

Supplementary Table 6: ICD-10 codes used to define mental illness, and types of mental illness, in the Swedish register population. 8

Supplementary Table 7: ATC codes used to identify psychiatric medication in the Swedish register population. 8

Supplementary Table 8: Distribution of sociodemographic variables in the total included COVIDMENT study population, and in each participating cohort, presented as N (%) or mean [SD]. 9

Supplementary Table 9: Uptake of COVID-19 vaccination in each included COVIDMENT cohort, overall and by presence of any mental illness diagnosis, presented as N (%). 11

Supplementary Table 10: Measurements of heterogeneity (I^2 index) from the overall and sex-stratified meta-analysis in the COVIDMENT study population. 13

Supplementary Table 11: Sensitivity model results (pooled prevalence ratio [PR] (95% CI)), according to the presence of any mental illness diagnosis, in the included COVIDMENT study population. 13

Supplementary Table 12: Uptake of COVID-19 vaccination by type of mental illness diagnosis and prescribed psychiatric medication use, in the included Swedish register population, presented as N (%). 14

Supplementary Table 13: Stratified results (prevalence ratio (95% CI)) from multivariable modified Poisson regression models performed in the Swedish register population, using 'any mental illness' and 'any medication' as exposure variables, and 'first dose of COVID-19 vaccination by 30th September 2021' and 'second dose of a COVID-19 vaccine by 30th November 2021' as outcome variables. 15

Supplementary Figure 1: Flow chart of COVIDMENT study population. 16

Supplementary Figure 2: Prevalence ratio (PR) and 95% CI of (A) first dose of a COVID-19 vaccine by 30th September 2021 in A1) females, and A2) males, (B) first dose of a COVID-19 vaccine by 18th February 2022 in B1) females, and B2) males, (C) second dose of a COVID-19 vaccine by 18th February 2022 in C1) females, and C2) males, according to the presence of any mental illness diagnosis, anxiety symptoms or depressive symptoms, in the included COVIDMENT study population. 17

Supplementary References 19

Supplementary Table 1: Ethical approvals obtained for each included COVIDMENT cohort study.

Cohort	Ethical Approval
EstBB cohorts	Estonian Committee on Bioethics and Human Research (1.1–12/1277 and 1.1–12/3454), Data release application (6-7/GI/27785) from the EstBB
C-19 Resilience	National Bioethics Committee (NBC no. 20– 073, 21–071) as well as the National Data Protection Authority
MAP-19	Regional Committee for Medical Research Ethics, reference number: 125510
MoBa	Regional Committees for Medical and Health Research Ethics (127708/14140/20138)
CovidLife	East of Scotland Research Ethics Service (EoSRES)
Omtanke2020	Ethical approval no. 2020–01785

EstBB cohorts (EstBB-C19 = The Estonian Biobank COVID-19 Cohort; EstBB-EHR = The Estonian Biobank electronic health records); C19-Resilience = The Icelandic COVID-19 National Resilience Cohort; MAP-19 = The Norwegian COVID-19, Mental Health and Adherence Project; MoBa = The Norwegian Mother, Father and Child Cohort Study

Supplementary Table 2: Key COVID-19 vaccination dates for countries included in the COVIDMENT study population.

Country	Date of first COVID-19 vaccination	Date at which all adults offered at least one dose of a COVID-19 vaccine
Estonia	January 2021[1]	May 2021[2]
Iceland	December 2020[3]	June 2021[4]
Norway	December 2020[5]	March 2021[6]
Scotland	December 2020[7]	June 2021[8]
Sweden	27th December 2020[9]	August- September 2021*[10-12]

*dependent on region of residence

Supplementary Table 3: ICD-10 codes used to define mental illness (in the EstBB-C19 and EstBB-EHR cohorts) and physical comorbidity status (in the EstBB-EHR cohort).

**Variable**	**Condition**	**ICD-10 codes**
Any mental illness		F00-F99
Physical comorbidity status	Hypertension	I10
	Heart disease	I00-I99 (excluding I10)
	Lung disease	J12-J18, J21-J22, J40-J47, J60-J70, J80-J84, E84.0, Q26.8, Q89.3, M05.1
	Chronic renal failure	N17-N19
	Cancer	C00-C97
	Diabetes	E10-E14
	Immunological conditions	B17-B40, D69.0, D69.3, D80-D89, E05.0, E06.3, E10-E14, G04.0, G04.8, G05.8, G35, G36.0-G36.1, G36.8-G36.9, G37.2-G37.5, G37.8-G37.9,G61.0, G70.0, H46.9, L10.0, L10.2, L12.0-L12.3, L12.8-L12.9, M05.0-M05.3, M05.8-M05.9, M06.0-M06.4, M06.8-M06.9, M08.0, M08.2-M08.4, M08.8-M08.9, M31.0, M32, M35.0, N08.5

Supplementary Table 4: Timing of variable definitions in the participating COVIDMENT cohorts.

Variable	Cohort	Timing of variable measurement
**Exposure variables**		
Diagnosis of any mental illness	EstBB-C19	Diagnosis from HIF bills (2004 - 26th December 2020)
	EstBB-EHR	Diagnosis from HIF bills (2004 - 26th December 2020)
	C-19 Resilience	Baseline questionnaire (24th April 2020-29th December 2020)
	MAP-19	Participants asked about mental illness diagnosis prior to the start of the COVID-19 pandemic in the following follow-up questionnaires: 24th October-12th November 2021, 2nd-14th January 2022, 6th-27th March 2022
	MoBa	For females: 1999-2008, 2007-2017; for males: 1999-2008, 2015<br>Responses were collected from the first questionnaire collected at recruitment (during pregnancy) between 1999-2009. Additional cases were collected from self-reported mental illnesses in questionnaires responded to when the children were 8 years old (between 2007 and 2017) for females (mothers), and in 2015 for males (fathers).<br>Participants could answer ”yes” (defined as mental illness diagnosis) or give no response.
	CovidLife	Baseline questionnaire (COVIDLife1: 17th April -7th June 2020)
	Omtanke2020	Baseline questionnaire (9th June 2020-26th December 2020)
Anxiety symptoms	EstBB-C19	C-19 questionnaire (10th May 2020 – 26th December 2020)
	EstBB-EHR	NA
	C-19 Resilience	Baseline questionnaire and monthly follow-up questionnaires (24th April 2020-29th December 2020)
	MAP-19	Baseline questionnaire (31st March-7th April 2020)
	MoBa	Biweekly follow-up questionnaires (11th May 2020-24th May 2020)
	CovidLife	Baseline questionnaire (COVIDLife1: 17th April -7th June 2020), second questionnaire (COVIDLife2: 21st July - 16th August 2020)
	Omtanke2020	Baseline and monthly follow-up questionnaires (9th June 2020-26th December 2020)
Depressive symptoms	EstBB-C19	C-19 questionnaire (10th May 2020 – 26th December 2020)
	EstBB-EHR	NA
	C-19 Resilience	Baseline questionnaire and monthly follow-up questionnaires (24th April 2020-29th December 2020)
	MAP-19	Baseline questionnaire (31st March-7th April 2020)
	MoBa	Biweekly follow-up questionnaires (10th June 2020-23rd June 2020)
	CovidLife	Baseline questionnaire (COVIDLife1: 17th April -7th June 2020), second questionnaire (COVIDLife2: 21st July - 16th August 2020)
	Omtanke2020	Baseline and monthly follow-up questionnaires (9th June 2020-26th December 2020)
**Outcome variables**		
First dose of a COVID-19 vaccine by 30th September 2021	EstBB-C19	e-Health Record registry immunization notices
	EstBB-EHR	e-Health Record registry immunization notices
	C-19 Resilience	Monthly follow-up questionnaires (27th May 2021-28th August 2021)
	MAP-19	Follow-up questionnaire (4th July-1st August 2021)
	MoBa	Biweekly follow-up questionnaires (2nd February 2021-30th September 2021)

	CovidLife	Scotland National COVID-19 vaccination data (8th December 2020 – 30th September 2021)
	Omtanke2020	Monthly follow-up questionnaires (27th Dec 2020-30th September 2021)
First dose of a COVID-19 vaccine by 18th February 2022	EstBB-C19	e-Health Record registry immunization notices
	EstBB-EHR	e-Health Record registry immunization notices
	C-19 Resilience	NA
	MAP-19	Follow-up questionnaire (2nd-14th January 2022)
	MoBa	Biweekly follow-up questionnaires (2nd February 2021-18th February 2022)
	CovidLife	Scotland National COVID-19 vaccination data (8th December 2020 – 30th September 2021)
	Omtanke2020	Annual follow-up questionnaire (1st December 2021-18th February 2022)
Second dose of a COVID-19 vaccine by 18th February 2022	EstBB-C19	e-Health Record registry immunization notices
	EstBB-EHR	e-Health Record registry immunization notices
	C-19 Resilience	NA
	MAP-19	Follow-up questionnaire (2nd-14th January 2022)
	MoBa	Biweekly follow-up questionnaires (2nd February 2021-18th February 2022)
	CovidLife	Scotland National COVID -19 vaccination data (8th December 2020 – 18th February 2022)
	Omtanke2020	Annual follow-up questionnaire (1st December 2021-18th February 2022)
**Covariates**		
Sociodemographic covariates (age, sex)	EstBB-C19	Based on national personal identification number drawn at recruitment
	EstBB-EHR	Based on national personal identification number drawn at recruitment
	C-19 Resilience	Baseline questionnaire (24th April 2020-29th December 2020)
	MAP-19	Baseline questionnaire (31st March 2020-7th April 2020)
	MoBa	First MoBa questionnaires filled out by females (mothers) and males (fathers) during pregnancy (recruitment 1999-2009)
	CovidLife	Baseline questionnaire (COVIDLife1: 17th April -7th June 2020)
	Omtanke2020	Baseline questionnaire (9th June 2020-26th December 2020)
Smoking status	EstBB-C19	C-19 questionnaire (10th May 2020 – 26th December 2020)
	EstBB-EHR	Baseline questionnaire completed upon joining EstBB (up to 26th December 2020)
	C-19 Resilience	Baseline questionnaire and monthly follow-up questionnaires (24th April 2020-29th December 2020)
	MAP-19	NA
	MoBa	Biweekly COVID-19 follow-up questionnaires (10th June 2020-23rd June 2020)
	CovidLife	Baseline questionnaire (COVIDLife1: 17th April -7th June 2020)
	Omtanke2020	Baseline questionnaire (9th June 2020-26th December 2020)
Previous COVID-19 infection	EstBB-C19	C-19 questionnaire (10th May 2020 – 26th December 2020)
	EstBB-EHR	e-Health Record registry (up to 26th December 2020)
	C-19 Resilience	Baseline questionnaire and monthly follow-up questionnaires (24th April 2020-29th December 2020)
	MAP-19	Baseline questionnaire (31st March 2020-7th April 2020)

	MoBa	Baseline COVID-19 questionnaire and biweekly COVID-19 follow-up questionnaires (31st March 2020-19th December 2020)
	CovidLife	Electronic Communication of Surveillance in Scotland (ECOSS) data on COVID-19 infections (29th January 2020 – 8th December 2020)
	Omtanke2020	Baseline and monthly follow-up questionnaires (9th June 2020-26th December 2020)
Physical comorbidity status	EstBB-C19	C-19 questionnaire (10th May 2020 – 26th December 2020)
	EstBB-EHR	Diagnosis from HIF bills (2004 - 26th December 2020)
	C-19 Resilience	Baseline questionnaire (24th April 2020-29th December 2020)
	MAP-19	NA
	MoBa	For chronic renal failure and immunological conditions: biweekly COVID-19 follow-up questionnaires (5th-18th December 2020)<br>For other conditions: baseline COVID-19 questionnaire (31st March 2020-12th May 2020)
	CovidLife	Baseline questionnaire (17th April -7th June 2020)
	Omtanke2020	Baseline questionnaire (9th June 2020-26th December 2020)

NA: not applicable (data no available in cohort)

EstBB-C19 = The Estonian Biobank COVID-19 Cohort; EstBB-EHR = The Estonian Biobank electronic health records; C19-Resilience = The Icelandic COVID-19 National Resilience Cohort; MAP-19 = The Norwegian COVID-19, Mental Health and Adherence Project; MoBa = The Norwegian Mother, Father and Child Cohort Study

Supplementary Table 5: National average Containment and Health Index from the Oxford COVID-19 Government Response Tracker (OxCGRT) between January 2020-September 2021, and resulting sub-group analysis category

Country	Average Containment and Health Index*	Sub-group analysis category
Sweden	48.79	Nordic
Norway	47.20	
Iceland	44.98	
UK	57.30	Non-Nordic
Estonia	38.54	

*calculated using data from the OxCGRT[13]

Supplementary Table 6: ICD-10 codes used to define mental illness, and types of mental illness, in the Swedish register population.

**Mental Illness**	**ICD-10 codes**
Any mental illness	F10-F19, F20-F29, F30-F33, F40-F41, F43
Substance use disorder (not including tobacco and alcohol)	F11-F19 (excluding F17)
Alcohol use disorder	F10
Tobacco use disorder	F17
Psychotic disorders (schizophrenia and non-affective psychotic disorders)	F20-F29
Bipolar disorder	F30, F31
Depression	F32, F33
Anxiety	F40, F41
Stress-related disorders	F43

Supplementary Table 7: ATC codes used to identify psychiatric medication in the Swedish register population.

**Psychiatric medication**	**ATC codes**
Any psychiatric medication	N06A, N05B, N05C, N05A
Antidepressants	N06A
Anxiolytics	N05B
Hypnotics/sedatives	N05C
Antipsychotics	N05A

Supplementary Table 8: Distribution of sociodemographic variables in the total included COVIDMENT study population, and in each participating cohort, presented as N (%) or mean [SD].

Variables	EstBB-C19 (Estonia) (n=5,633)	EstBB-EHR (Estonia) (n=183,332)	C-19 Resilience (Iceland) (n=10,417)	MAP-19 (Norway) (n=3,894)	MoBa (Norway) (n=102,811)	CovidLife (Scotland) (n=4,760)	Omtanke2020 (Sweden) (n=14,451)	Total (N=325,298)
**Sex**								
Female	4,240 (75.3%)	120,436 (65.7%)	7,115 (68.3%)	3,002 (77.1%)	61,973 (60.3%)	2,989 (62.8%)	12,057 (83.4%)	211,812 (65.1%)
Male	1,393 (24.7%)	62,896 (34.3%)	3,193 (30.7%)	883 (22.7%)	40,838 (39.7%)	1,767 (37.1%)	2,394 (16.6%)	113,364 (34.9%)
Other	0 (0.0%)	0 (0.0%)	15 (0.1%)	0 (0.0%)	0 (0.0%)	0 (0.0%)	0 (0.0%)	15 (0.0%)
Missing	0 (0.0%)	0 (0.0%)	94 (0.9%)	9 (0.2%)	0 (0.0%)	4 (0.1%)	0 (0.0%)	107 (0.0%)
**Age group, years**								
18-29	589 (10.5%)	23,751 (13.0%)	427 (4.1%)	1,552 (39.9%)	0 (0.0%)	43 (0.9%)	1,399 (9.7%)	27,761 (8.5%)
30-39	1,426 (25.3%)	40,308 (22.0%)	909 (8.7%)	888 (22.8%)	8,860 (8.6%)	358 (7.5%)	2,053 (14.2%)	54,802 (16.9%)
40-49	1,337 (23.7%)	37,637 (20.5%)	1,727 (16.6%)	660 (16.9%)	65,942 (64.2%)	587 (12.3%)	2,462 (17.0%)	110,352 (33.9%)
50-59	1,259 (22.4%)	33,892 (18.5%)	2,736 (26.2%)	465 (11.9%)	26,549 (25.8%)	1,092 (22.9%)	3,335 (23.1%)	69,328 (21.3%)
60-69	727 (12.9%)	26,667 (14.5%)	2,943 (28.3%)	259 (6.7%)	1,252 (1.2%)	1,662 (34.9%)	2,918 (20.2%)	36,428 (11.2%)
70 or above	295 (5.2%)	21,077 (11.5%)	1,675 (16.1%)	70 (1.8%)	67 (0.1%)	1,011 (21.3%)	2,284 (15.8%)	26,479 (8.1%)
Missing	0 (0.0%)	0 (0.0%)	0 (0.0%)	0 (0.0%)	141 (0.1%)	7 (0.2%)	0 (0.0%)	148 (0.1%)
Mean [SD] age, years	46.3 [13.6]	48.1 [16.2]	56.1 [13.4]	36.9 [13.8]	46.5 [5.3]	59.4 [12.1]	52.3 [15.6]	48.0 [2.9]
**Previous COVID-19 infection**								
Yes	912 (16.2%)	3,772 (2.0%)	327 (3.1%)	5 (0.1%)	2,007 (2.0%)	83 (1.7%)	634 (4.4%)	7,740 (2.4%)
No	4,721 (83.8%)	67,946 (37.1%)	10,090 (96.9%)	3,889 (99.9%)	100,804 (98.0%)	4,677 (98.3%)	13,817 (95.6%)	205,944 (63.3%)
Missing	0 (0.0%)	111,614 (60.9%)	0 (0.0%)	0 (0.0%)	0 (0.0%)	0 (0.0%)	0 (0.0%)	111,614 (34.3%)
**Smoking status**								
Yes	773 (13.7%)	36,035 (19.7%)	1,486 (14.3%)	0 (0.0%)	20,297 (19.8%)	257 (5.4%)	1,932 (13.4%)	60,780 (18.7%)
No	4,542 (80.6%)	130,074 (70.9%)	8,918 (85.6%)	0 (0.0%)	81,967 (79.7%)	4,414 (92.7%)	12,496 (86.5%)	242,411 (74.5%)
Missing	318 (5.7%)	17,223 (9.4%)	13 (0.1%)	3,894 (100.0%)	547 (0.5%)	89 (1.9%)	23 (0.1%)	22,107 (6.8%)
**Chronic physical conditions**								
0	2,574 (45.7%)	68,730 (37.5%)	5,735 (55.1%)	0 (0.0%)	81879 (79.6%)	3,546 (74.5%)	9,250 (64.0%)	171,714 (52.8%)
1	1,805 (32.0%)	55,229 (30.1%)	3,099 (29.7%)	0 (0.0%)	17169 (16.7%)	972 (20.4%)	3,353 (23.2%)	81,627 (25.1%)
≥2	775 (13.8%)	59,373 (32.4%)	1,485 (14.3%)	0 (0.0%)	3763 (3.7%)	217 (4.6%)	1,218 (8.4%)	66,831 (20.5%)

Missing	479 (8.5%)	0 (0.0%)	98 (0.9%)	3,894 (100.0%)	0 (0.0%)	25 (0.5%)	630 (4.4%)	5,126 (1.6%)

EstBB-C19 = The Estonian Biobank COVID-19 Cohort; EstBB-EHR = The Estonian Biobank electronic health records; C19-Resilience = The Icelandic COVID-19 National Resilience Cohort; MAP-19 = The Norwegian COVID-19, Mental Health and Adherence Project; MoBa = The Norwegian Mother, Father and Child Cohort Study

Supplementary Table 9: Uptake of COVID-19 vaccination in each included COVIDMENT cohort, overall and by presence of any mental illness diagnosis, presented as N (%).

	**Uptake of first dose of a COVID-19 vaccine by 30th September 2021**			**Uptake of first dose of a COVID-19 vaccine by 18th February 2022**			**Uptake of second dose of a COVID-19 vaccine by 18th February 2022**		
	Yes	No	Total	Yes	No	Total	Yes	No	Total
**EstBB-C19 (Estonia)**	5,108 (90.7%)	525 (9.3%)	5,633 (100.0%)	5,243 (93.1%)	390 (6.9%)	5,633 (100.0%)	4,950 (94.4%)	294 (5.6%)	5,244 (100.0%)
Any mental illness	2,796 (90.0%)	311 (10.0%)	3,107 (100.0%)	2,870 (92.4%)	237 (7.6%)	3,107 (100.0%)	2,708 (94.4%)	162 (5.6%)	2,870 (100.0%)
No mental illness	2,312 (91.5%)	214 (8.5%)	2,526 (100.0%)	2,373 (93.9%)	153 (6.1%)	2,526 (100.0%)	2,242 (94.4%)	132 (5.6%)	2,374 (100.0%)
Missing	0 (0.0%)	0 (0.0%)	0 (0.0%)	0 (0.0%)	0 (0.0%)	0 (0.0%)	0 (0.0%)	0 (0.0%)	0 (0.0%)
**EstBB-EHR (Estonia)**	150,387 (82.0%)	32,945 (18.0%)	183,332 (100.0%)	157,983 (86.2%)	25,349 (13.8%)	183,332 (100.0%)	143,877 (94.4%)	8,469 (5.6%)	152,346 (100.0%)
Any mental illness	76,855 (80.7%)	18,353 (19.3%)	95,208 (100.0%)	81,038 (85.1%)	14,170 (14.9%)	95,208 (100.0%)	73,634 (94.2%)	4,541 (5.8%)	78,175 (100.0%)
No mental illness	73,532 (83.4%)	14,592 (16.6%)	88,124 (100.0%)	76,945 (87.3%)	11,179 (12.7%)	88,124 (100.0%)	70,243 (94.7%)	3,928 (5.3%)	74,171 (100.0%)
Missing	0 (0.0%)	0 (0.0%)	0 (0.0%)	0 (0.0%)	0 (0.0%)	0 (0.0%)	0 (0.0%)	0 (0.0%)	0 (0.0%)
**C-19 Resilience (Iceland)**	9,117 (87.5%)	1,300 (12.5%)	10,417 (100.0%)	NA	NA	NA	NA	NA	NA
Any mental illness	2,460 (85.0%)	435 (15.0%)	2,895 (100.0%)	NA	NA	NA	NA	NA	NA
No mental illness	6,446 (88.5%)	837 (11.5%)	7,283 (100.0%)	NA	NA	NA	NA	NA	NA
Missing	211 (88.3%)	28 (11.7%)	239 (100.0%)	NA	NA	NA	NA	NA	NA
**MAP-19 (Norway)**	2,529 (78.3%)	702 (21.7%)	3,231 (100.0%)	2,523 (97.2%)	74 (2.8%)	2,597 (100.0%)	2,482 (98.4%)	41 (1.6%)	2,523 (100.0%)
Any mental illness	425 (73.9%)	150 (26.1%)	575 (100.0%)	503 (95.8%)	22 (4.2%)	525 (100.0%)	492 (97.8%)	11 (2.2%)	503 (100.0%)
No mental illness	1,736 (79.6%)	446 (20.4%)	2,182 (100.0%)	2,020 (97.5%)	52 (2.5%)	2,072 (100.0%)	1,990 (98.5%)	30 (1.5%)	2,020 (100.0%)
Missing	368 (77.6%)	106 (22.4%)	474 (100.0%)	0 (0.0%)	0 (0.0%)	0 (0.0%)	0 (0.0%)	0 (0.0%)	0 (0.0%)
**MoBa (Norway)**	91,424 (89.5%)	10,704 (10.5%)	102,128 (100.0%)	94,500 (91.9%)	8,311 (8.1%)	102,811 (100.0%)	91,442 (96.8%)	3,058 (3.2%)	94,500 (100.0%)
Any mental illness	13,688 (89.0%)	1,696 (11.0%)	15,384 (100.0%)	14,163 (91.4%)	1,333 (8.6%)	15,496 (100.0%)	13,658 (96.4%)	505 (3.6%)	14,163 (100.0%)
No mental illness	77,736 (89.6%)	9,008 (10.4%)	86,744 (100.0%)	80,337 (92.0%)	6,978 (8.0%)	87,315 (100.0%)	77,784 (96.8%)	2,553 (3.2%)	80,337 (100.0%)
Missing	0 (0.0%)	0 (0.0%)	0 (0.0%)	0 (0.0%)	0 (0.0%)	0 (0.0%)	0 (0.0%)	0 (0.0%)	0 (0.0%)

**CovidLife (Scotland)**	4,477 (94.1%)	283 (5.9%)	4,760 (100.0%)	4,484 (94.2%)	276 (5.8%)	4,760 (100.0%)	4,461 (100.0%)	1 (0.0%)	4,462 (100.0%)
Any mental illness	1,151 (93.6%)	79 (6.4%)	1,230 (100.0%)	1,151 (93.6%)	79 (6.4%)	1,230 (100.0%)	1,145 (100.0%)	0 (0.0%)	1,145 (100.0%)
No mental illness	3,296 (94.2%)	203 (5.8%)	3,499 (100.0%)	3,303 (94.4%)	196 (5.6%)	3,499 (100.0%)	3,290 (100.0%)	1 (0.0%)	3,291 (100.0%)
Missing	30 (96.8%)	1 (3.2%)	31 (100.0%)	30 (96.8%)	1 (3.2%)	31 (100.0%)	26 (100.0%)	0 (0.0%)	26 (100.0%)
**Omtanke2020 (Sweden)**	4,939 (92.7%)	387 (7.3%)	5,326 (100.0%)	14,154 (97.9%)	297 (2.1%)	14,451 (100.0%)	5,227 (98.1%)	102 (1.9%)	5,329 (100.0%)
Any mental illness	1,666 (91.9%)	147 (8.1%)	1,813 (100.0%)	4,230 (97.4%)	112 (2.6%)	4,342 (100.0%)	1,783 (98.2%)	32 (1.8%)	1,815 (100.0%)
No mental illness	3,116 (93.1%)	232 (6.9%)	3,348 (100.0%)	9,634 (98.3%)	170 (1.7%)	9,804 (100.0%)	3,281 (98.0%)	68 (2.0%)	3,349 (100.0%)
Missing	157 (95.2%)	8 (4.8%)	165 (100.0%)	290 (95.1%)	15 (4.9%)	305 (100.0%)	163 (98.8%)	2 (1.2%)	165 (100.0%)

Supplementary Table 10: Measurements of heterogeneity (I^2 index) from the overall and sex-stratified meta-analysis in the COVIDMENT study population.

Variable	I^2 index from meta-analysis								
	Any mental illness diagnosis			Anxiety symptoms			Depressive symptoms		
	Overall study population	Females only	Males only	Overall study population	Females only	Males only	Overall study population	Females only	Males only
First dose of a COVID-19 vaccine by 30th September 2021	91.4% (*p*<0.0001)	85.5% (*p*<0.0001)	85.6% (*p*<0.0001)	60.7% (*p*=0.0153)	56.0% (*p*=0.0337)	0.0% (*p*=0.7864)	63.0% (*p*=0.0071)	67.2% (*p*=0.0018)	0.0% (*p*=0.8428)
First dose of a COVID-19 vaccine by 18th February 2022	80.0% (*p*<0.0001)	76.7% (*p*<0.0001)	72.7 (*p*<0.0001)	73.7% (*p*=0.0034)	72.6% (*p*=0.0082)	30.4% (*p*=0.2867)	73.4% (*p*=0.0067)	78.1% (*p*=0.0030)	2.2% (*p*=0.4151)
Second dose of a COVID-19 vaccine by 18th February 2022	60.7% (*p*=0.0843)	31.4% (*p*=0.1483)	5.7% (*p*=0.3042)	2.0% (*p*=0.4837)	47.0% (*p*=0.1643)	49.6% (*p*=0.1454)	53.4% (*p*=0.1185)	59.4% (*p*=0.0770)	0.0% (*p*=0.4911)

The I^2 index is calculated by dividing the result from Cochran's Q test and its corresponding degrees of freedom by the Q-value. No adjustments were made for multiple comparisons.

Supplementary Table 11: Sensitivity model results (pooled prevalence ratio [PR] (95% CI)), according to the presence of any mental illness diagnosis, in the included COVIDMENT study population.

	Sensitivity analysis 1		Sensitivity analysis 2		Sensitivity analysis 3		
	Pooled PR (95% CI)	I^2	Pooled PR (95% CI)	I^2	Country category	Pooled PR (95% CI)	I^2
**Outcome**							
First dose of a COVID-19 vaccine by 30th September 2021	0.99 (0.96-1.01)	91.3% (*p*=0.0010)	0.99 (0.98-1.00)	87.1% (*p*<0.0001)	Nordic	0.99 (0.96-1.01)	91.3% (*p*=0.0010)
					Non-Nordic	0.98 (0.96-1.00)	84.0% (*p*=0.0004)
First dose of a COVID-19 vaccine by 18th February 2022	0.99 (0.99-1.00)	18.2% (*p*=0.3251)	0.99 (0.98-1.00)	76.8% (*p*=0.0020)	Nordic	0.99 (0.99-1.00)	18.2% (*p*=0.3251)
					Non-Nordic	0.98 (0.97-0.99)	67.4% (*p*=0.0357)
Second dose of a COVID-19 vaccine by 18th February 2022	NA		1.00 (0.99-1.00)	22.5% (*p*=0.1348)	NA		

Sensitivity analysis 1: exclusion of cohorts which used electronic health records for the definition of exposure and/or outcome variables; sensitivity analysis 2: exclusion of individuals with any chronic physical conditions; sensitivity analysis 3: sub-group analysis, based on the average national Oxford COVID-19 Government Response Tracker (OxCGRT) Containment and Health Index

NA: not applicable (sensitivity analysis 1 and 3 could not be run for the 'second dose of a COVID-19 vaccine by 18th February 2022' as only two cohorts could be included in the (sub-group) meta-analysis)

The I^2 index is calculated by dividing the result from Cochran's Q test and its corresponding degrees of freedom by the Q-value. No adjustments were made for multiple comparisons.

Supplementary Table 12: Uptake of COVID-19 vaccination by type of mental illness diagnosis and prescribed psychiatric medication use, in the included Swedish register population, presented as N (%).

	First dose of a COVID-19 vaccine by 30th September 2021			Second dose of a COVID-19 vaccine by 30th November 2021		
	**Yes**	**No**	**Total**	**Yes**	**No**	**Total**
**Type of mental illness**						
Substance use disorder*	35,607 (61.5%)	22,312 (38.5%)	57,919 (100.0%)	32,346 (90.8%)	3,261 (9.2%)	35,607 (100.0%)
Alcohol use disorder	59,850 (78.4%)	16,504 (21.6%)	76,354 (100.0%)	57,156 (95.5%)	2,694 (4.5%)	59,850 (100.0%)
Tobacco use disorder	21,536 (84.8%)	3,868 (15.2%)	25,404 (100.0%)	21,037 (97.7%)	499 (2.3%)	21,536 (100.0%)
Psychotic disorders	34,341 (74.8%)	11,593 (25.2%)	45,934 (100.0%)	33,183 (96.6%)	1,158 (3.4%)	34,341 (100.0%)
Bipolar disorder	41,625 (84.0%)	7,927 (16.0%)	49,552 (100.0%)	40,309 (96.8%)	1,316 (3.2%)	41,625 (100.0%)
Depression	137,243 (80.7%)	32,742 (19.3%)	169,985 (100.0%)	132,040 (96.2%)	5,202 (3.8%)	137,242 (100.0%)
Anxiety	156,427 (77.5%)	45,344 (22.5%)	201,771 (100.0%)	149,715 (95.7%)	6,712 (4.3%)	156,427 (100.0%)
Stress-related disorders	86,160 (76.2%)	26,901 (23.8%)	113,061 (100.0%)	82,635 (95.9%)	3,525 (4.1%)	86,160 (100.0%)
**Type of psychiatric medication**						
Antidepressants	1,127,619 (87.7%)	158,523 (12.3%)	1,286,142 (100.0%)	1,104,282 (97.9%)	23,336 (2.1%)	1,127,618 (100.0%)
Anxiolytics	673,869 (85.9%)	110,972 (14.1%)	784,841 (100.0%)	658,987 (97.8%)	14,881 (2.2%)	673,868 (100.0%)
Hypnotics/sedatives	940,546 (88.3%)	125,222 (11.8%)	1,065,768 (100.0%)	922,633 (98.1%)	17,911 (1.9%)	940,544 (100.0%)
Antipsychotics	157,254 (81.3%)	36,252 (18.7%)	193,506 (100.0%)	152,536 (97.0%)	4,718 (3.0%)	157,254 (100.0%)

*Not including alcohol and tobacco use disorders.

Supplementary Table 13: Stratified results (prevalence ratio (95% CI)) from multivariable modified Poisson regression models performed in the Swedish register population, using 'any mental illness' and 'any medication' as exposure variables, and 'first dose of COVID-19 vaccination by 30th September 2021' and 'second dose of a COVID-19 vaccine by 30th November 2021' as outcome variables.

	**Prevalence Ratio (95% CI)**			
	**First dose of a COVID-19 vaccine by 30th September 2021**		**Second dose of a COVID-19 vaccine by 30th November 2021**	
	**Any mental illness**	**Any medication**	**Any mental illness**	**Any medication**
**Sex**				
Male	0.97 (0.97-0.98)	1.02 (1.02-1.02)	0.99 (0.98-0.99)	1.00 (1.00-1.00)
Female	1.00 (1.00-1.00)	1.03 (1.03-1.03)	0.99 (0.99-0.99)	1.00 (1.00-1.00)
**Chronic physical condition**				
0	0.99 (0.99-0.99)	1.03 (1.03-1.03)	0.99 (0.99-0.99)	1.00 (1.00-1.00)
≥1	0.97 (0.97-0.97)	1.01 (1.01-1.01)	0.99 (0.99-0.99)	1.00 (1.00-1.00)

No adjustments were made for multiple comparisons.

Supplementary Figure 1: Flow chart of COVIDMENT study population.

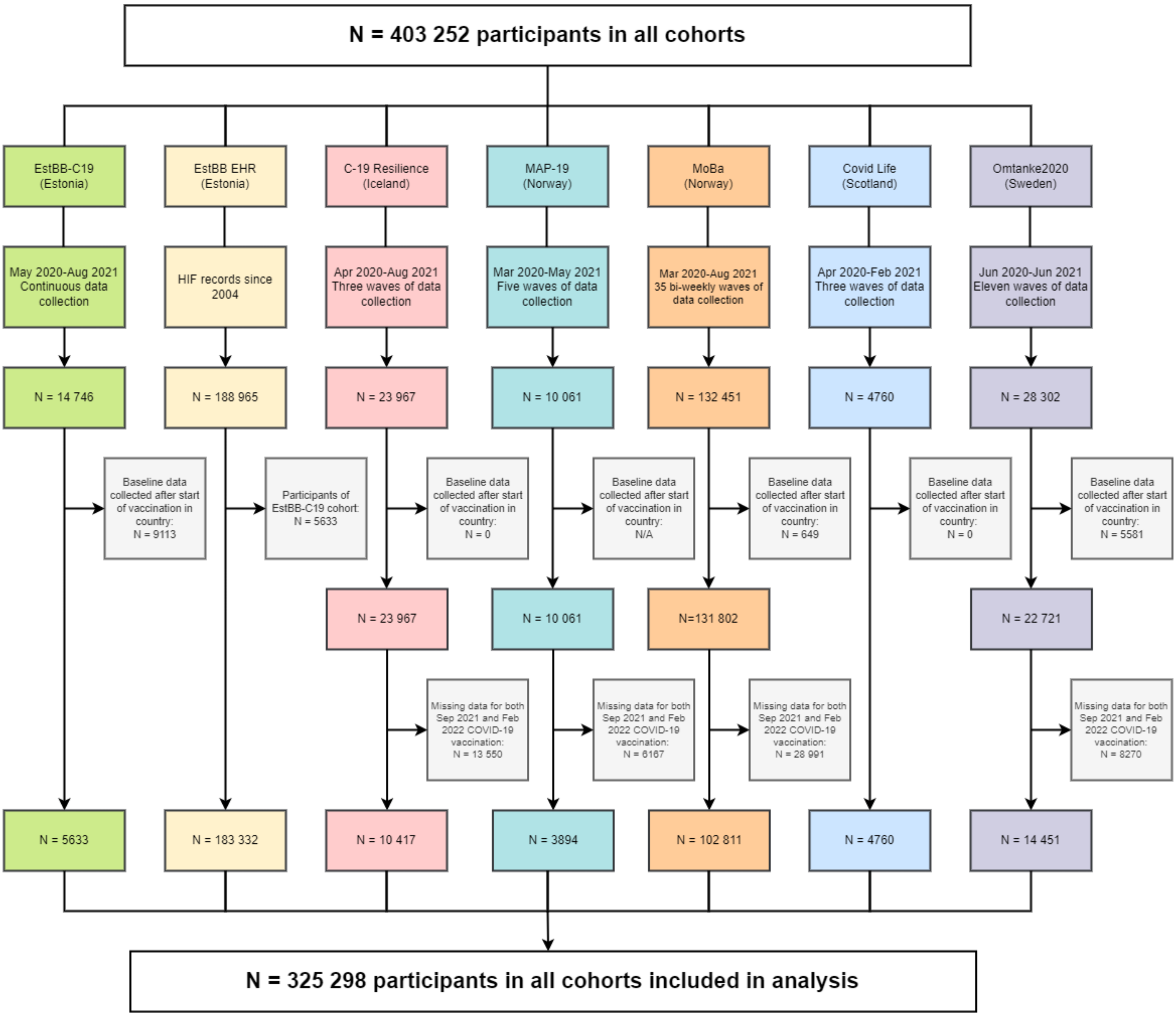

EstBB-C19 = The Estonian Biobank COVID-19 Cohort; EstBB-EHR = The Estonian Biobank electronic health records; C19-Resilience = The Icelandic COVID-19 National Resilience Cohort; MAP-19 = The Norwegian COVID-19, Mental Health and Adherence Project; MoBa = The Norwegian Mother, Father and Child Cohort Study). places. Only CovidLife participants with linked vaccination data from Generation Scotland were included in the study population.

Supplementary Figure 2: Prevalence ratio (PR) and 95% CI of (A) first dose of a COVID-19 vaccine by 30th September 2021 in A1) females, and A2) males, (B) first dose of a COVID-19 vaccine by 18th February 2022 in B1) females, and B2) males, (C) second dose of a COVID-19 vaccine by 18th February 2022 in C1) females, and C2) males, according to the presence of any mental illness diagnosis, anxiety symptoms or depressive symptoms, in the included COVIDMENT study population.

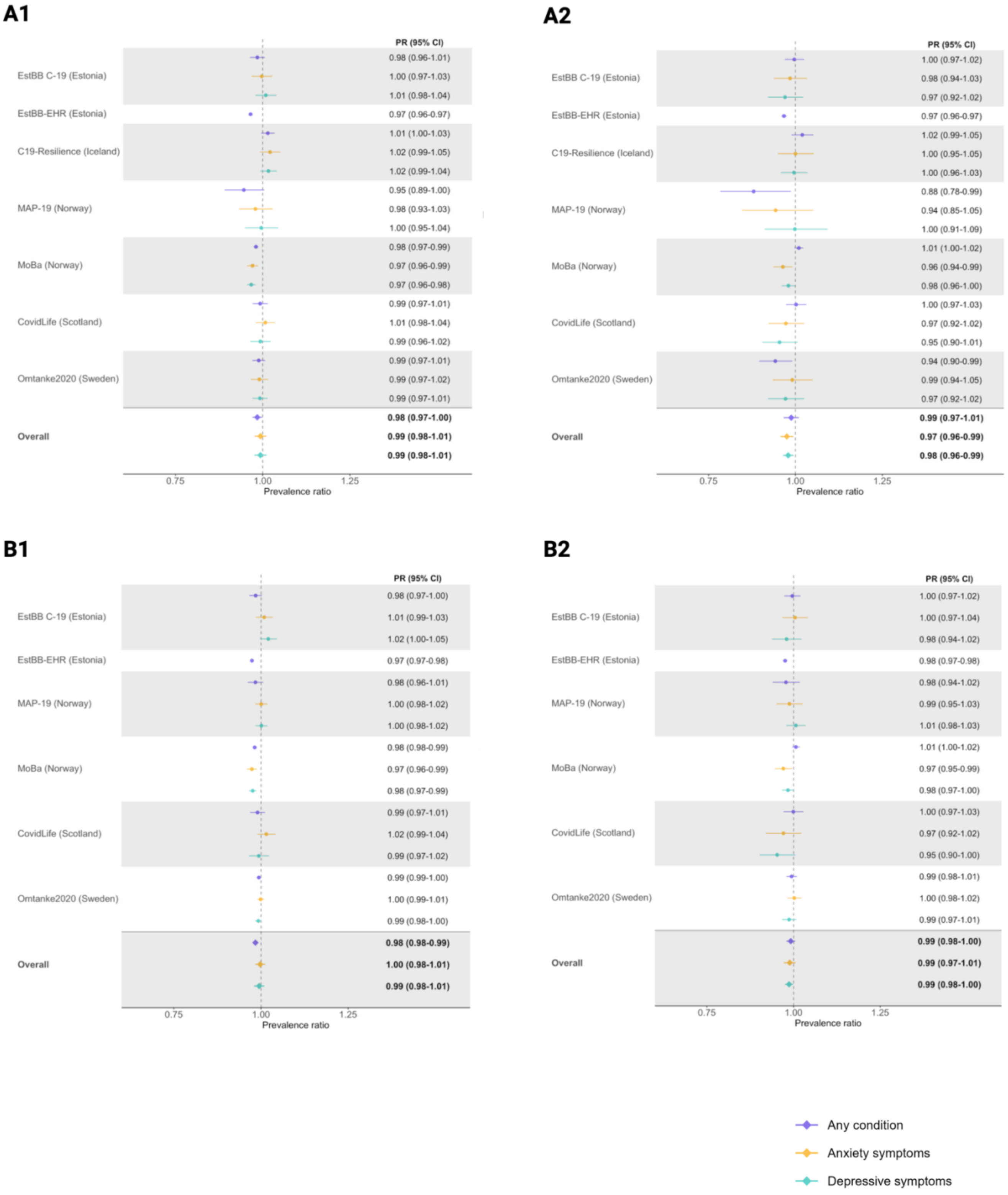

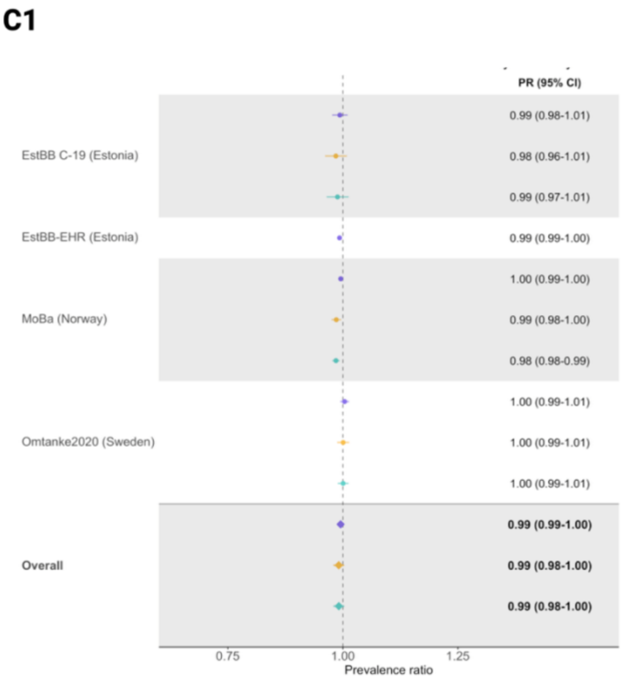

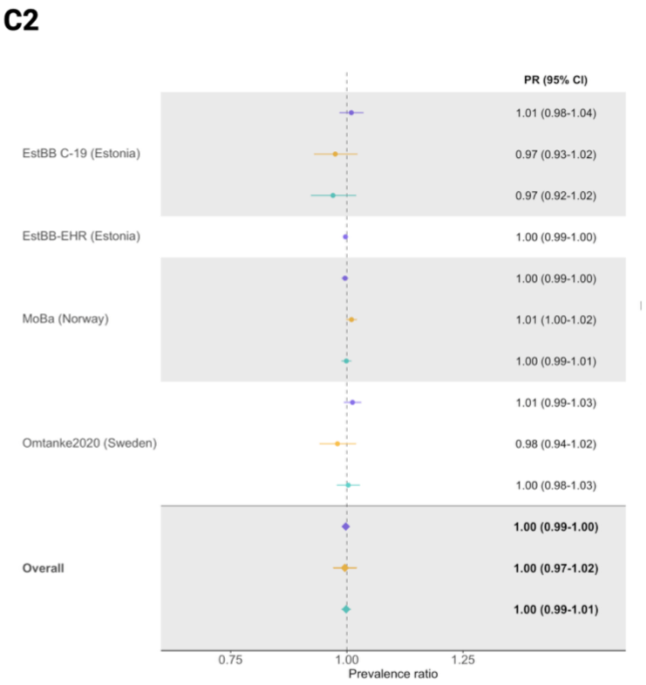

Data are presented as PR with 95% CIs (horizontal lines), rounded to 2 decimal places. Cohort-specific estimates are adjusted for age, previous COVID-19 infection, smoking, and physical comorbidity status (except MAP-19 models, which are adjusted for age and previous COVID-19 infection only). The 'overall' estimates are derived from the random effects meta-analyses of the cohort-specific estimates.

EstBB cohorts (EstBB-C19 = The Estonian Biobank COVID-19 Cohort; EstBB-EHR = The Estonian Biobank electronic health records); C19-Resilience = The Icelandic COVID-19 National Resilience Cohort; MAP-19 = The Norwegian COVID-19, Mental Health and Adherence Project; MoBa = The Norwegian Mother, Father and Child Cohort Study).

Total N (any mental illness diagnosis; anxiety symptoms; depressive symptoms) = (A1) 191,004; 72,623; 70,877; (A2) 104,306; 40,670; 39,436; (B1) 191,827; 73,026; 71,223; (B2) 102,820; 38,999; 37,726; (C1) 158,749; 60,392; 58,792; (C2) 87,294; 34,438; 33,423.

Supplementary References

1. Uusküla A, Kalda R, Solvak M, Jürisson M, Käärik M, Fischer K, Keis A, Raudvere U, Vilo J, Peterson H, Käärik E. The 1st year of the COVID-19 epidemic in Estonia: a population-based nationwide sequential/consecutive cross-sectional study. Public Health. 2022 Apr;205:150-6
2. Republic of Estonia: Ministry of Social Affairs. Vaccination against COVID-19 will be gradually available to anyone during May. 2021. Accessed: 10 October 2022. Available from: https://www.sm.ee/en/news/vaccination-against-covid-19-will-be-gradually-available-anyone-during-may
3. The Directorate of Health. Iceland's response. 2022. Accessed: 1 Jan 2023. Available from: https://www.covid.is/sub-categories/iceland-s-response
4. Government of Iceland. COVID-19: Iceland removes all domestic COVID restrictions. 2021. Accessed: 10 October 2022. Available from: https://www.government.is/news/article/2021/06/25/COVID-19-Iceland-removes-all-domestic-COVID-restrictions/
5. Skjesol I, Tritter JQ. The Norwegian way: COVID-19 vaccination policy and practice. Health Policy and Technology. 2022 Jun;11(2%):100635
6. Norwegian Institute of Public Health (NIPH%). AstraZeneca vaccine recommended for all over 18 years of age. 2021. Accessed: 10 October 2022]. Available from: https://www.fhi.no/en/archive/covid-19-archive/covid-19---archived-news-2021/march/astrazeneca-vaccine-recommended-for-all-over-18-years-of-age/
7. Scottish Government. First COVID-19 vaccinations in Scotland take place. 2020. Accessed 2 Jan 2023. Available from: https://www.gov.scot/news/first-covid-19-vaccinations-in-scotland-take-place/
8. Scottish Government. First doses booked in for all adults ahead of schedule. 2021. Accessed: 10 October 2022. Available from: https://www.gov.scot/news/first-doses-booked-in-for-all-adults-ahead-of-schedule/
9. Krisinformation.se. Covid-19 vaccination begins on 27 December. 2020 10 October 2022]; Available from: https://www.krisinformation.se/en/news/2020/december/vaccination-27-december
10. Sveriges Kommuner och Regioner [SKR]. Regionernas planering avseende vaccinering mot covid-19, delrapport 6 [The planning regarding vaccination against Covid-19 for regions, report 6]. 2021. Accessed: 10 October 2021. Available from: https://skr.se/download/18.5bb54e0c179a302981232fd/1621980314035/Regionernas_planer%20ing_%20vaccinering_covid-19_delrapport%206.pdf
11. Krisinformation.se. Vecka 26 2021. Accessed: 10 October 2022. 2021. Available from: https://www.krisinformation.se/om-krisinformation/for-myndigheter-och-andra-aktorer/omvarldsbevakning/20212/vecka-26-2021
12. Folkhälsomyndigheten. När hände vad under pendemin? Accessed: 31 July 2024. Available from: https://www.folkhalsomyndigheten.se/smittskydd-beredskap/utbrott/utbrottsarkiv/covid-19-pandemin-2019-2023/nar-hande-vad-under-pandemin/
13. Hale T, Angrist N, Goldszmidt R, Kira B, Petherick A, Phillips T, et al. A global panel database of pandemic policies (Oxford COVID-19 Government Response Tracker). Nature human behaviour. 2021;5(4):529-38.
