# Supplementary material for: Mental illness and COVID-19 vaccination: a multinational investigation of observational & register-based data
Source: Nat Commun. 2024 Sep 26;15:8124. doi: 10.1038/s41467-024-52342-1 (PMC11427681; doi:10.1038/s41467-024-52342-1)
Supplement: Supplementary file 3 — Reporting Summary [file 41467_2024_52342_MOESM3_ESM.pdf]

nature portfolio

Corresponding author(s): Mary M Barker

Last updated by author(s): 26/8/24

Reporting Summary

Nature Portfolio wishes to improve the reproducibility of the work that we publish. This form provides structure for consistency and transparency in reporting. For further information on Nature Portfolio policies, see our Editorial Policies and the Editorial Policy Checklist.

Statistics

For all statistical analyses, confirm that the following items are present in the figure legend, table legend, main text, or Methods section.

n/a	Confirmed	
☐	☒	The exact sample size (*n*) for each experimental group/condition, given as a discrete number and unit of measurement
☐	☒	A statement on whether measurements were taken from distinct samples or whether the same sample was measured repeatedly
☐	☒	The statistical test(s) used AND whether they are one- or two-sided<br>*Only common tests should be described solely by name; describe more complex techniques in the Methods section.*
☐	☒	A description of all covariates tested
☐	☒	A description of any assumptions or corrections, such as tests of normality and adjustment for multiple comparisons
☐	☒	A full description of the statistical parameters including central tendency (e.g. means) or other basic estimates (e.g. regression coefficient) AND variation (e.g. standard deviation) or associated estimates of uncertainty (e.g. confidence intervals)
☐	☒	For null hypothesis testing, the test statistic (e.g. *F*, *t*, *r*) with confidence intervals, effect sizes, degrees of freedom and *P* value noted<br>*Give P values as exact values whenever suitable.*
☒	☐	For Bayesian analysis, information on the choice of priors and Markov chain Monte Carlo settings
☒	☐	For hierarchical and complex designs, identification of the appropriate level for tests and full reporting of outcomes
☐	☒	Estimates of effect sizes (e.g. Cohen's *d*, Pearson's *r*), indicating how they were calculated

Our web collection on statistics for biologists contains articles on many of the points above.

Software and code

Policy information about availability of computer code

Data collection	No software was used
Data analysis	STATA (version 17.0) and R (version 4.3.0) were used.

For manuscripts utilizing custom algorithms or software that are central to the research but not yet described in published literature, software must be made available to editors and reviewers. We strongly encourage code deposition in a community repository (e.g. GitHub). See the Nature Portfolio guidelines for submitting code & software for further information.

Data

Policy information about availability of data

All manuscripts must include a data availability statement. This statement should provide the following information, where applicable:

- Accession codes, unique identifiers, or web links for publicly available datasets
- A description of any restrictions on data availability
- For clinical datasets or third party data, please ensure that the statement adheres to our policy

The raw datasets are not available for sharing due to privacy policies and regulations in the respective countries.

Research involving human participants, their data, or biological material

Policy information about studies with human participants or human data. See also policy information about sex, gender (identity/presentation), and sexual orientation and race, ethnicity and racism.

Reporting on sex and gender	In the COVIDMENT analysis, self-reported sex was included as a covariate, and adjusted for in all models. Analyses stratified by sex were also performed. The register-based analysis used sex data from the Swedish registers for adjustment and stratification.
Reporting on race, ethnicity, or other socially relevant groupings	Data unavailability in some of the COVIDMENT studies meant that several socially relevant groupings (e.g. income status, education level, ethnicity) could not be included as a covariate in the COVIDMENT analysis. However, sociodemographic data is available in the registers and therefore models using register-based data were adjusted for the available variables (highest educational attainment, cohabitation status, and income). Ethnicity data is not available in the Swedish registers.
Population characteristics	Population characteristics for the COVIDMENT study population and the Swedish register study population are displayed in full in Tables 1 and 3, respectively. Briefly, in the COVIDMENT study population, 65.1% of participants were female, and the mean age of participants was 48 years. In the register-based study population, 50.0% of individuals were female and the mean age was 49.9 years.
Recruitment	For the register-based part of the study, Swedish nationwide register data was used. The included COVIDMENT cohort studies used different recruitment strategies (recruiting from established cohorts, self-recruitment via social media). The potential for selection bias in the cohort studies is described in the discussion section.
Ethics oversight	Swedish register data use was approved by Swedish Ethical Review Authority (2020-01800 with subsequent amendments). Ethical approvals for all included COVIDMENT cohorts were obtained from regional or national ethics committees, and are shown in detail in Supplementary Table 1.

Note that full information on the approval of the study protocol must also be provided in the manuscript.

Field-specific reporting

Please select the one below that is the best fit for your research. If you are not sure, read the appropriate sections before making your selection.

☐ Life sciences ☒ Behavioural & social sciences ☐ Ecological, evolutionary & environmental sciences

For a reference copy of the document with all sections, see nature.com/documents/nr-reporting-summary-flat.pdf

Behavioural & social sciences study design

All studies must disclose on these points even when the disclosure is negative.

Study description	A quantitative cohort-based study was conducted.
Research sample	The COVIDMENT study population included participants from seven cohort studies conducted in Sweden, Norway, Estonia, Iceland, and Scotland. All of the COVIDMENT cohort studies had a higher proportion of female vs. male participants. The mean ages of participants in all cohorts were between 36-60 years. The Swedish register data included all individuals living in Sweden during the study period (27th December 2020-30th November 2021). Of the register study population, 50% were male and the mean age was 49.9 years.
Sampling strategy	The included cohort studies recruited participants using different strategies: recruiting from established cohorts and/or self-recruitment via social media. No sample size calculation was performed; analyses were conducted using all eligible participants from the cohort studies, in addition to all individuals living in Sweden during the study period.
Data collection	The COVIDMENT cohort study questionnaires were completed online by participants. For the majority of the cohort studies, self-report measures were completed by participants to ascertain covariates, exposures (previous diagnosis of any mental illness, anxiety symptoms, depressive symptoms), and outcomes (COVID-19 vaccination uptake). EstBB-EHR and CovidLife used electronic health records to collect some or all of the relevant data. Register data is managed by public agency representatives and clinicians. The included cohort studies conducted various data collection waves during 2020 and 2021. Exposure variables were defined using data collected before the initiation of COVID-19 vaccination in the cohort's respective country. Outcome variables were conducted using data collected before the defined end dates for each outcome.
Timing	The exposure variables (mental illness) were identified before the initiation of COVID-19 vaccination in each country. The outcome variables (COVID-19 vaccination) were identified between the start of vaccination in each country and the specific end dates for each outcome, with the reasons for the chosen end dates specified in the manuscript.
Data exclusions	All exclusion criteria were pre-established and explained in detail in the manuscript. Of the total study populations included (cohort study population: N=325,293, Swedish register population: N=8,080,234) all individuals with nonmissing data were included in the analyses. The specific number of individuals included in each analytical model are displayed in the Figure legends.
Non-participation	Supplementary Figure 1 shows in detail the number of participants who were eligible for inclusion in the current study from the

Non-participation	COVIDMENT cohort studies. Of the 403,252 individuals included in the participating COVIDMENT cohort studies, 325,298 individuals met the eligibility criteria for the present study. For the register-based analysis, indivdiauls from the total Swedish population were dropped if they didn't meet the specific study eligibility criteria, resulting in a study population of 8,080,234 individuals.
Randomization	As the study was observational, no randomisation was used.

Reporting for specific materials, systems and methods

We require information from authors about some types of materials, experimental systems and methods used in many studies. Here, indicate whether each material, system or method listed is relevant to your study. If you are not sure if a list item applies to your research, read the appropriate section before selecting a response.

Materials & experimental systems

n/a	Involved in the study
☒	☐ Antibodies
☒	☐ Eukaryotic cell lines
☒	☐ Palaeontology and archaeology
☒	☐ Animals and other organisms
☒	☐ Clinical data
☒	☐ Dual use research of concern
☒	☐ Plants

Methods

n/a	Involved in the study
☒	☐ ChIP-seq
☒	☐ Flow cytometry
☒	☐ MRI-based neuroimaging

Plants

Seed stocks	*Report on the source of all seed stocks or other plant material used. If applicable, state the seed stock centre and catalogue number. If plant specimens were collected from the field, describe the collection location, date and sampling procedures.*
Novel plant genotypes	*Describe the methods by which all novel plant genotypes were produced. This includes those generated by transgenic approaches, gene editing, chemical/radiation-based mutagenesis and hybridization. For transgenic lines, describe the transformation method, the number of independent lines analyzed and the generation upon which experiments were performed. For gene-edited lines, describe the editor used, the endogenous sequence targeted for editing, the targeting guide RNA sequence (if applicable) and how the editor was applied.*
Authentication	*Describe any authentication procedures for each seed stock used or novel genotype generated. Describe any experiments used to assess the effect of a mutation and, where applicable, how potential secondary effects (e.g. second site T-DNA insertions, mosiacism, off-target gene editing) were examined.*
